# Household survey data of adoption of improved varieties and management practices in rice production, Ecuador

**DOI:** 10.1016/j.dib.2018.04.019

**Published:** 2018-04-10

**Authors:** Diego Marin, Mayra Orrego-Varon, Fernando Yanez, Luis Mendoza, Maria Alejandra Garcia, Jennifer Twyman, Robert Andrade, Ricardo Labarta

**Affiliations:** a International Centre for Tropical Agriculture (CIAT), Cali, Colombia; b National Agricultural Research Institute (INIAP), Quito, Ecuador

## Abstract

This article provides a description of an agricultural household survey data of rice growers collected in Ecuador between October 2014 and March 2015. The household survey was implemented using a structured questionnaire administered among 1028 households in the main rice production areas of Ecuador (i.e. Guayas, Los Rios, Manabi, and El Oro provinces). Information collected was provided by household heads (male or female) and included household and plot level data. The survey information includes household socio-demographic characteristics (e.g. age, education, gender, main economic activity, etc.), farm characteristics (e.g. farm land size, assets ownership, other crops planted, etc.), rice management practices (e.g. variety and input use, production costs, etc.), and rice production and utilization (e.g. yields, prices, sales, etc.). Additional socio-economic context variables were also recorded such as government subsidies to rice production, participation in rural organizations, and food security related questions. The dataset contains a total of 6288 variables among numeric, categorical and string variables. The dataset is shared publicly on the Harvard dataverse site and provide access to questionnaires, the complete data and a brief report.

**Specifications table**

**Table:** 

Subject area	Agricultural sciences
More specific subject area	Adoption of improved rice varieties and management practices
Type of data	Categorical, string and numeric variables
How data was acquired	Household surveys through face to face interviews
Data format	STATA (dta) files and CSV files in raw format
Experimental factors	Sample consisted of 1028 rice growers selected randomly in main rice production areas in Ecuador in a cross-sectional survey
Experimental features	Factors that facilitate or limit the adoption of rice innovations
Data source location	84 farm communities distributed in Guayas, Los Rios, Manabi, and El Oro provinces of Ecuador
Data accessibility	The data accompanying this article can be found online at: https://dataverse.harvard.edu/dataset.xhtml?persistentId=doi:10.7910/DVN/DX3F4T

**Value of the data**

• The dataset with detailed plot level information can be used to characterize rice farming systems in Ecuador and parametrize and develop rice crop models to evaluate biotic and abiotic effects on production.
• The dataset can be used to estimate the level of adoption of different rice varieties and agronomic practices in Ecuador, and analyze factors influencing adoption of agricultural innovations in the rice sector. This analysis could support a better targeting of agricultural extension programs.
• The dataset contains key variables to estimate productivity and livelihood impacts of using agricultural innovations (e.g. improved varieties, better management practices, input use, public policies) that would inform agricultural policies in the rice sector.

## 1 Data

The dataset described in this article was collected in the coastal region of Ecuador (Guayas, Los Rios, Manabi, and El Oro provinces) between November 2014 and April 2015. Ecuador is one of the most important rice producing countries in Latin America and where improved rice technologies have been widely promoted [1]. The dataset was collected collaboratively between the International Center for Tropical Agriculture (CIAT) and the National Agricultural Research Institute of Ecuador (INIAP). Table 1 describes key socioeconomics characteristics of the respondents of the survey.

**Table 1:** Socioeconomics characteristics of the 1028 respondents of the survey.

Feature	Characteristics	Value^a^
Gender	Men	952 (92.79%)
	Female	74 (7.21%)
Average age	Years	52.35 (13.4)
Average years of schooling	Years	6.01 (3.82)
Agricultural experience	Years	26.91 (15.22)
Average agricultural land	Hectares	5.9 (9.1)
Average rice area	Hectares	4.8 (7.9)
Input subsidy program	Participants	450 (43.86%)
	Non participants	576 (56.14%)

a Mean for continuous variables and proportions for dichotomous variables.

The dataset recorded information about household characteristics (i.e. age, gender, household composition, marital status, education, and main economic activity), rice production (i.e. seeds usage, area planted, soil, fertilizer and input management, irrigation systems, and harvest, usage and market access), land ownership and tenure, other crops farmed, production decision making disaggregated by gender (e.g. seed usage, plot management, etc.), productive assets endowment, measurements of poverty level (using the Progress out of Poverty Index) and food security (using consumption during the last 24 h and week), access to credit and public services, and climate change perceptions.

**Fig. 1:**
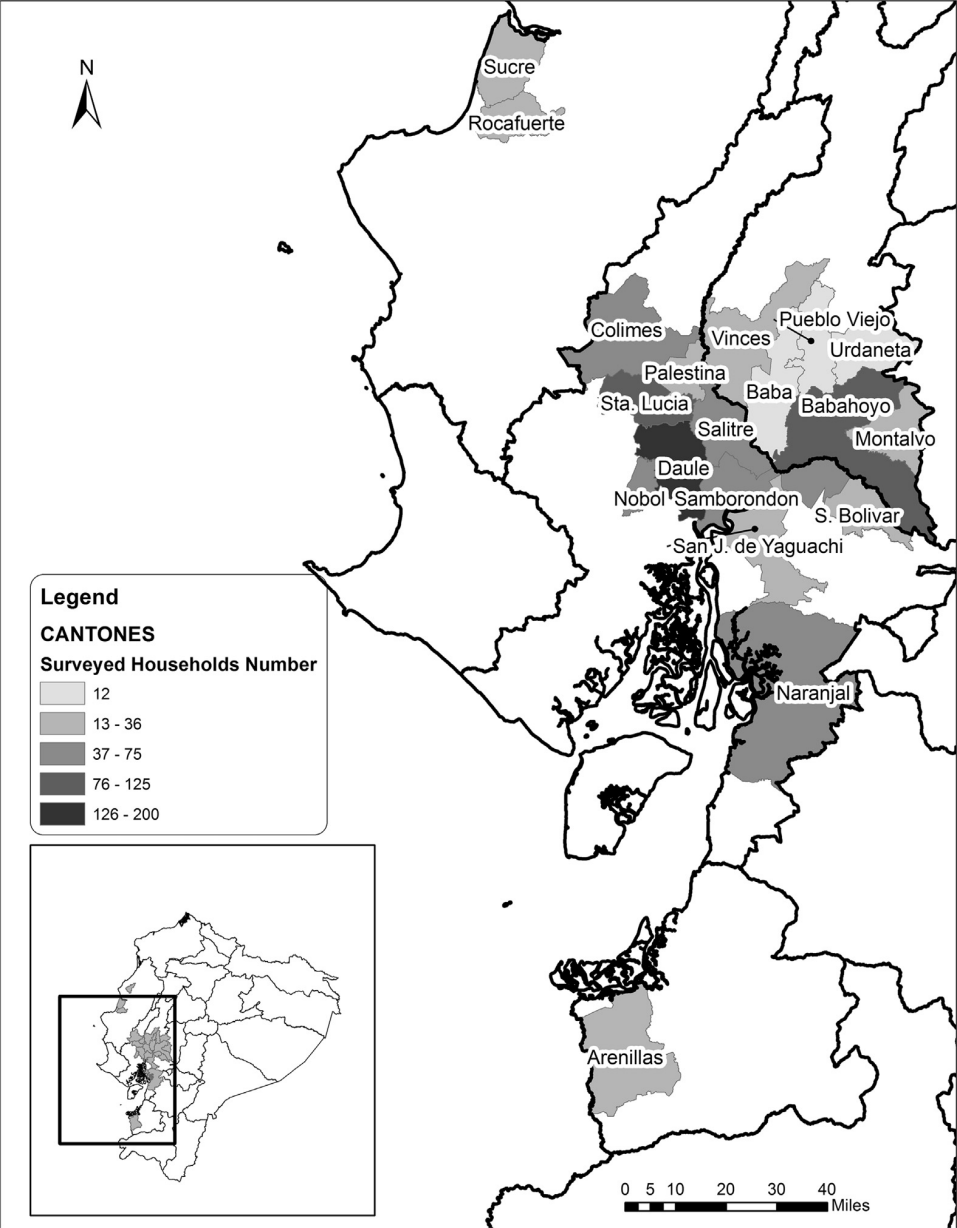
Map showing parishes in Ecuador where 84 communities were sampled].

The survey was collected by interviewing the farm household head (male or female). In addition to the complete data, a variable dictionary describing labels and the questionnaire used for data collection are available in the Harvard dataverse. Identification variables such as farmer's name, GPS (Global Positioning System) coordinates, and community names were removed and they are available only upon request (Fig. 1).

## 2 Experimental design, materials and methods

Following [2], a two-stage and stratified random sampling approach was used to select 1028 rice farm households in Ecuador. The sampling framework was design to be nationally representative of the target population, which was the rice producers in the main rice producing areas of Ecuador. The sample frame represented 97% of total rice production in the country. In the first stage a total of 84 farm communities were selected randomly using rice area planted at province and canton^1^ levels to build an associated probability of each community of being selected [3]. (Fig. 1). In the second stage twelve rice producers were randomly selected within each community. Given the interest to understand the role of gender into rice production in Ecuador, the sampling design aimed to interview at least three female heads in each community. Nevertheless, due to the cultural context of the Ecuadorian rural areas, it was only possible to interview female headed households in 7.2 percent of the total sample. The dataset was collected using paper questionnaires. Field work was carried by four teams of three enumerators and a coordinator with multiple backgrounds (i.e. agronomy, sociology and economics). Data was entered and verified using CSPro (Census and Survey Processing System) before being exported to Stata 13.1 and CSV (Comma-Separated Values*)* formats.

## Footnotes

1 In Ecuador provinces are the next smaller administrative unit of the country. Ecuador has 24 provinces and within each province there are cantons and within them there are communities.
